# Low-order many-body interactions determine the local structure of liquid water†
†Electronic supplementary information (ESI) available: Brief overview of the many-body expansion of the total energy and details about the functional form adopted by MB-pol. Additional analyses of many-body effects in (H_2_O)_*n*_ clusters with *n* = 1–6. Comparisons between radial distribution functions (RDFs) calculated from PIMD simulations with the MB-XC PEFs introduced in this study and the corresponding *ab initio* XC functionals. See DOI: 10.1039/c9sc03291f


**DOI:** 10.1039/c9sc03291f

**Published:** 2019-07-26

**Authors:** Marc Riera, Eleftherios Lambros, Thuong T. Nguyen, Andreas W. Götz, Francesco Paesani

**Affiliations:** a Department of Chemistry and Biochemistry , University of California , San Diego , La Jolla , California 92093 , USA; b San Diego Supercomputer Center , University of California , San Diego , La Jolla , California 92093 , USA; c Materials Science and Engineering , University of California , San Diego , La Jolla , California 92093 , USA . Email: fpaesani@ucsd.edu

## Abstract

Despite its apparent simplicity, water displays unique behavior across the phase diagram which is strictly related to the ability of the water molecules to form dense, yet dynamic, hydrogen-bond networks that continually fluctuate in time and space. The competition between different local hydrogen-bonding environments has been hypothesized as a possible origin of the anomalous properties of liquid water. Through a systematic application of the many-body expansion of the total energy, we demonstrate that the local structure of liquid water at room temperature is determined by a delicate balance between two-body and three-body energies, which is further modulated by higher-order many-body effects. Besides providing fundamental insights into the structure of liquid water, this analysis also emphasizes that a correct representation of two-body and three-body energies requires sub-chemical accuracy that is nowadays only achieved by many-body models rigorously derived from the many-body expansion of the total energy, which thus hold great promise for shedding light on the molecular origin of the anomalous behavior of liquid water.

## Abstract 2

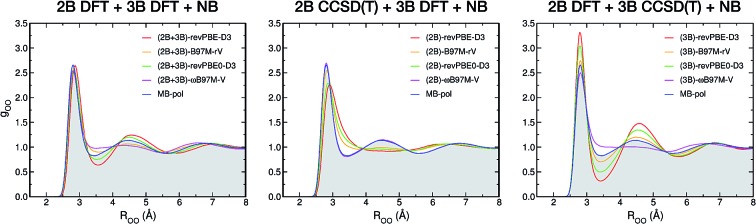
Two-body and three-body energies, modulated by higher-body terms and nuclear quantum effects, determine the structure of liquid water and require sub-chemical accuracy that is achieved by the MB-pol model but not by existing DFT functionals.

## 1 Introduction

Covering 71% of the Earth's surface and making up more than two-thirds of the human body weight, water plays an essential role in life which cannot be overemphasized.^1^ After decades of research, there is now no doubt that the unique properties of water cannot simply be explained in terms of a three-atom, 10-electron molecule, but are rather due to the ability of the water molecules to form a dense, yet flexible, hydrogen-bond (H-bond) network in which both number and strength of H-bonds continually fluctuate. While temperature and pressure variations cause smooth changes in the local properties of simple liquids, this is not the case in liquid water where similar variations modify the equilibrium of different H-bonding environments, resulting in drastic changes in the local structure and, consequently, in both thermodynamic and dynamical properties.^2^ The competition between two different local H-bonding environments, commonly defined as high-density liquid (HDL) and low-density liquid (LDL) phases, has been suggested as a possible origin of the anomalous properties of liquid water.^3^

As already recognized by Frank and Wen in their picture of liquid water consisting of “flickering clusters of hydrogen-bonded molecules”,^4^ the relative stability of different H-bonding arrangements in liquid water results from the delicate interplay of many-body effects that may either increase (cooperative effects) or decrease (anticooperative effects) the strength of the overall interaction relative to the sum of all pairwise contributions.^5^ These many-body interactions are further modulated by nuclear quantum effects.^6^,^7^ Many-body interactions in a system containing *N* water molecules can be rigorously defined through the corresponding many-body expansion (MBE) of the total energy (*E*_*N*_),^8^1
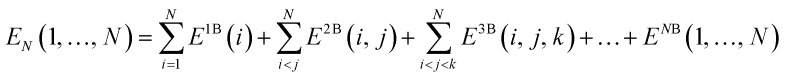
where *E*^1B^(*i*) corresponds to the one-body (1B) energy required to deform the *i*^th^ water molecule from its equilibrium geometry, and *E*^*n*B^(*i*, *j*, …, *n*) are the *n*-body (*n*B) energies defined recursively as2
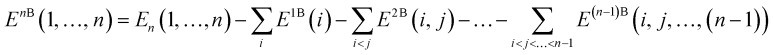



It has been shown that eqn (1) converges rapidly for water, with the 2B and 3B terms contributing, on average, ∼80% and 15–20%, respectively, and all remaining higher-body terms contributing ∼1%.^8^–^10^

Besides representing a rigorous theoretical framework for characterizing many-body interactions in liquid water, eqn (1) also provides an efficient computational route for developing analytical potential energy functions (PEFs) that express the total energy of a system composed of *N* water molecules as a sum of individual many-body terms.^11^ This has led to the advent of several many-body PEFs for water in which the individual many-body terms in eqn (1) are fitted to large sets of reference data calculated at the coupled cluster level of theory with single, double, and iterative triple excitations, *i.e.*, CCSD(T), the current “gold standard” for molecular interactions.^12^–^17^ Among existing many-body PEFs, it has been shown that the MB-pol PEF achieves great accuracy by integrating classical representations of many-body electrostatics, which correctly describe interactions at large molecular separations, with data-driven 2B and 3B terms represented by permutationally invariant polynomials (PIPs) that smoothly turn on as two and three water molecules, respectively, approach each other.^15^–^17^ The MB-pol 2B and 3B terms, which were fitted to large sets of CCSD(T) reference data calculated in the complete basis set (CBS) limit, effectively account for non-classical interactions (*e.g.*, Pauli repulsion, charge transfer and charge penetration) arising in regions where the electron densities of individual water molecules overlap.^18^

Building upon the theoretical framework provided by eqn (1) and the functional form adopted by the MB-pol PEF, we demonstrate that the local structure of liquid water is determined by a delicate balance between 2B and 3B interactions, which requires sub-chemical accuracy for a correct representation of the underlying H-bonding arrangements.

## 2 Methods

### 2.1 Electronic structure calculations

**Fig. 1:**
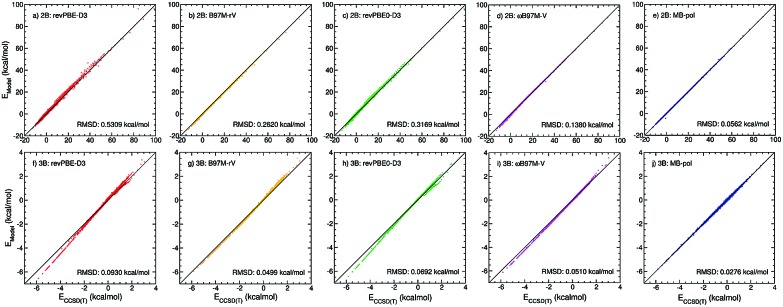
Correlation plots for 2B (top panels) and 3B (bottom panels) energies. Plotted on the *x* axes are CCSD(T)/CBS reference values calculated for 42 508 water dimers (ref. 15) and 12 347 water trimers (ref. 16), respectively. On the *y* axes are the corresponding energies calculated with revPBE-D3 (red, panels (a) and (f)), B97M-rV (yellow, panels (b) and (g)), revPBE0-D3 (green, panels (c) and (h)), *ω*B97M-V (magenta, panels (d) and (i)), and MB-pol (blue, panels (e) and (j)).

All CCSD(T) calculations presented in the main text were carried out using MOLPRO.^19^ The 2B energies shown in Fig. 1 of the main text were computed by extrapolating the values obtained with aug-cc-pVTZ and aug-cc-pVQZ basis sets supplemented with an additional set of (3s, 3p, 2d, 1f) midbond functions, with exponents equal to (0.9, 0.3, 0.1) for s and p orbitals, (0.6, 0.2) for d orbitals, and 0.3 for f orbitals, placed at the center of mass of each dimer configuration.^20^–^22^ The following two-point formula was used to extrapolate the energies to the CBS limit:^23^,^24^
3
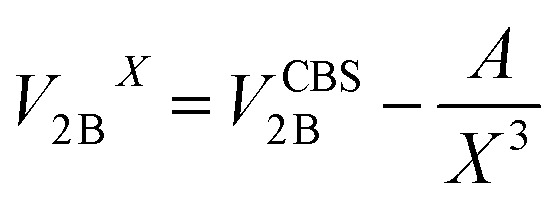
with cardinal numbers *X* = 3 and 4, accordingly. The Hartree–Fock energy was not extrapolated separately since it was close to the CBS limit for either value of *X*. The 3B energies were calculated at the CCSD(T) level of theory using the aug-cc-pVTZ basis set supplemented with the same set of midbond functions introduced above, which were placed at the center of mass of each trimer configuration, and were corrected for the basis set superposition error (BSSE) using the counterpoise method.^25^

The reference interaction energies for (H_2_O)_*n*_ clusters, with *n* = 2–6, were obtained using the MBE of the interaction energy^8^ according to the “stratified approximation many-body approach” (SAMBA) as described in ref. 26. Within SAMBA, the 2B and 3B energies were calculated as described above while all higher (>3B) contributions were computed with the CCSD(T)-F12 method using the VTZ-F12 basis sets.^27^–^29^ This method yields results close to the CBS values at lower computational cost than canonical CCSD(T) calculations with large basis sets.^30^,^31^ Cluster geometries for *n* = 2–3 where taken from ref. 32, those for *n* = 4–5 were taken from ref. 33, and those for *n* = 6 were taken from ref. 34. All DFT calculations with revPBE-D3,^35^ B97M-rV,^36^ revPBE0-D3,^37^ and *ω*B97M-V^38^ functionals were carried with the aug-cc-pVQZ basis set^20^ using Q-Chem.^39^

### 2.2 Path-integral molecular dynamics

Path-integral molecular dynamics (PIMD) simulations^40^–^43^ were carried out in the normal-mode representation to calculate both radial distribution functions, RDFs, and distributions of the tetrahedral order parameter, *P*(*q*_tet_). PIMD is based upon Feynman's formulation of statistical mechanics in terms of path integrals^44^ and exploits the isomorphism between the quantum partition function of a system of *N* particles and the classical partition function of a system consisting of *N* flexible ring polymers.^40^ By construction, PIMD enables the calculation of numerically exact structural and thermodynamic properties of quantum-mechanical systems.^45^ All PIMD simulations were carried out for a system consisting of 256 H_2_O molecules in a periodic cubic box in both canonical (NVT: constant number of molecules, volume, and temperature) and isobaric-isothermal (NPT: constant number of molecules, pressure, and temperature) ensembles, with *T* = 298.15 K and *P* = 1.0 atm. Each atom was represented by a ring polymer with 32 beads.^45^ In both ensembles, the equations of motion were propagated for 1 ns using the velocity-Verlet algorithm with a time step Δ*t* = 0.2 fs. The temperature was controlled *via* Nosé–Hoover chains (NHC) of four thermostats coupled to each degree of freedom.^46^ The NPT ensemble was generated according to the algorithm described in ref. 47. A radial atom–atom cutoff distance of 9.0 Å was applied to the nonbonded interactions and the Ewald sum was used to treat the long-range electrostatic interactions.^48^

### 2.3 Tetrahedral order parameter

The tetrahedral order parameters *q*_tet_ was calculated as^49^4
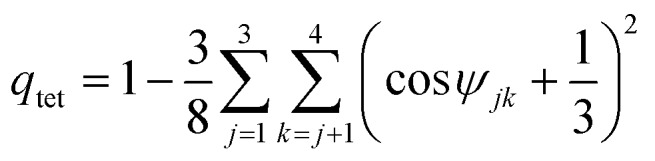
where *ψ*_*jk*_ is the angle between the oxygen atom of the central molecule and the oxygen atoms of the two closest molecules *j* and *k*, with *j*, *k* ≤ 4. As shown in ref. 49, *q*_tet_ = 1 for a perfect tetrahedral environment and *q*_tet_ = 0 for a completely random environment. The normalized probability distributions of the tetrahedral order parameter, *P*(*q*_tet_), were calculated from the corresponding PIMD simulations by averaging over all 32 beads.

## 3 Results

### 3.1 Many-body potential energy functions with *ab initio* accuracy

To investigate the role played by many-body interactions in determining the structure of liquid water we introduce a series of many-body PEFs that, as MB-pol, are rigorously derived from eqn (1), but, differently from MB-pol, are fitted to reference energies calculated at the density functional theory (DFT) level, using four representative exchange–correlation (XC) functionals belonging to different rungs across the Jacob's ladder of DFT approximations.^50^ Specifically, considering that the main contributions to the interaction energies between water molecules are associated with the 2B and 3B energies,^11^ and all many-body terms involving four or more water molecules are quantitatively described by MB-pol,^18^ we introduce here four families of many-body PEFs in which the original MB-pol 2B and 3B terms, originally fitted to CCSD(T)/CBS reference data, are replaced by analogous terms fitted to 2B and 3B energies calculated with the revPBE-D3 (rung 2),^35^,^51^ B97M-rV (rung 3),^36^ revPBE0-D3 (rung 4),^37^,^51^ and *ω*B97M-V (rung 4)^38^ functionals, while all other many-body terms of eqn (1) are represented as in MB-pol^15^,^16^ (see ESI† for a systematic analysis of many-body contributions to the interaction energies of water clusters).

Using the same dimer and trimer training sets adopted in the development of MB-pol,^15^,^16^ Fig. 1 shows correlation plots between 2B and 3B energies calculated with each of the four XC functionals and the corresponding CCSD(T)/CBS reference values.^15^,^16^ Also shown for comparison are the correlation plots for MB-pol. Three general observations can be made with respect to this analysis. First, the overall accuracy of the four XC functionals improves from revPBE-D3 (rung 2) to *ω*B97M-V (rung 4), with the notable exception of B97M-rV that outperforms revPBE0-D3 despite belonging to a lower rung. Second, all four functionals provide relatively better agreement with the CCSD(T)/CBS reference values for 2B than 3B energies. Third, MB-pol provides the smallest root-mean-square deviation (RMSD) from the CCSD(T)/CBS reference data for both 2B and 3B energies.

### 3.2 Many-body effects and the energetics of water clusters

**Fig. 2:**
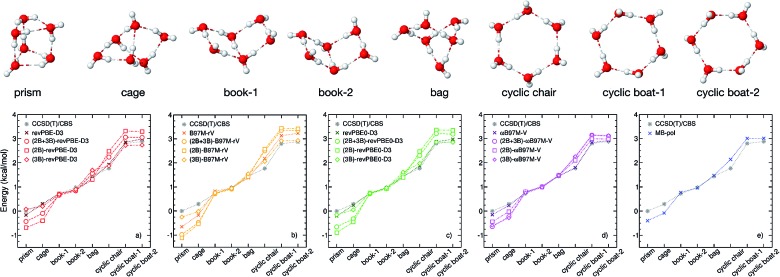
(Top) Geometries of the low-lying isomers of the water hexamer. (Bottom) Comparison of relative interaction energies for the low-lying isomers of the water hexamer calculated using the (2B+3B)-XC PEFs (open circles), (2B)-XC PEFs (open squares), and (3B)-XC PEFs (open diamonds) with XC = revPBE-D3 (red, panel (a)), B97M-rV (yellow, panel (b)), revPBE0-D3 (green, panel (c)), and *ω*B97M-V (magenta, panel (d)). The isomer geometries were taken from ref. 34. Also shown in each panel are the reference CCSD(T)/CBS values. Analogous comparison between MB-pol and CCSD(T)/CBS reference values taken from ref. 32 are shown in panel (e).

To investigate how the interplay between individual many-body effects determines both water structure and energetics, the DFT 2B and 3B energies shown in Fig. 1 are used in eqn (1) to develop three distinct many-body PEFs for each of the four XC functionals. These many-body PEFs, hereafter referred to as (MB)-XC PEFs (with XC = revPBE-D3, B97M-rV, revPBE0-D3, and *ω*B97M-V) adopt the same functional form as MB-pol but progressively replace the MB-pol 2B and 3B terms, which were fitted to CCSD(T)/CBS data, with analogous fits to the corresponding 2B and 3B energies calculated with the four XC functionals shown in Fig. 1. For each XC functional, the three distinct models are thus labeled as (2B+3B)-XC, (2B)-XC, and (3B)-XC to indicate that both 2B and 3B, only 2B, and only 3B terms are represented by fits to the corresponding data calculated with the specific XC functional, while all other terms are taken from MB-pol. Since the water hexamer exhibits three-dimensional structures reminiscent of the three-dimensional hydrogen-bonding arrangements in liquid water, the ability to reproduce the relative stability of the hexamer isomers is often used to assess the accuracy of water models. Fig. 2 shows that all three many-body PEFs constructed for each XC functional predict relative energies of the low-lying isomers of the water hexamer that are always within 1.0 kcal mol^–1^, commonly defined as “chemical accuracy”, of the corresponding values calculated fully *ab initio* with the same XC functional. This analysis thus provides evidence that all MB-XC PEFs are able to correctly reproduce many-body effects as described by the corresponding XC functional.

### 3.3 Many-body effects and the structure of liquid water

**Fig. 3:**
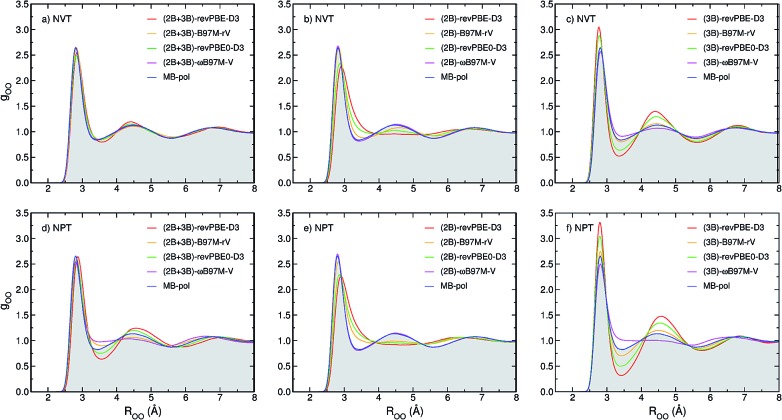
Comparison between oxygen–oxygen radial distribution functions (RDFs) of liquid water at ambient conditions derived from X-ray diffraction measurements (gray area) of ref. 52 and calculated from PIMD simulations carried out at 298.15 K in the NVT (panels a–c) and NPT (panels d–f) ensembles with the (2B+3B)-XC PEFs (panels a and d), (2B)-XC PEFs (panels b and e), and (3B)-XC PEFs (panels c and f) with XC = revPBE-D3 (red), B97M-rV (yellow), revPBE0-D3 (green), and *ω*B97M-V (magenta). Also shown in each panel are the corresponding RDFs calculated from PIMD simulations with MB-pol.

To determine how 2B and 3B contributions affect the structure of liquid water at ambient conditions, path-integral molecular dynamics (PIMD) simulations are carried out at 298.15 K in both NVT and NPT ensembles. Fig. 3 shows comparisons between the experimental oxygen–oxygen radial distribution function (RDF)^52^ and the corresponding RDFs calculated from PIMD simulations in the NVT (top panels) and NPT (bottom panels) ensembles using the three MB-XC PEFs developed for each of the four XC functionals. Also shown for reference are the corresponding MB-pol RDFs.^17^ Comparisons between the corresponding oxygen-hydrogen and hydrogen–hydrogen RDFs are reported in the ESI.† Fig. 3a demonstrates that, when the simulations are carried out in the NVT ensemble with the density fixed at the experimental value, all (2B+3B)-XC PEFs accurately reproduce the experimental O–O RDF, with only revPBE-D3 predicting a slightly over structured liquid. As discussed in the ESI,† the RDFs calculated with the (2B+3B)-XC PEFs using revPBE-D3, B97M-rV, and revPBE0-D3 are in agreement with those reported in the literature from *ab initio* PIMD simulations carried out with the corresponding functionals,^53^,^54^ which provides evidence for the ability of the present (2B+3B)-XC PEFs to faithfully reproduce the corresponding DFT results at a fraction of the cost associated with fully *ab initio* simulations. In this context, it should also be noted that small noticeable differences are seen in the comparison between the oxygen–oxygen RDF calculated with the (2B+3B)-revPBE-D3 PEF and the corresponding results from *ab initio* simulations with the bare revPBE-D3 functional.^53^ These differences are due to differences in how (2B+3B)-revPBE and revPBE-D3 represent 4B energies. While in (2B+3B)-revPBE the 4B energies are described as in MB-pol which, as shown in Fig. S4–S6 of the ESI,† provides excellent agreement with the CCSD(T)/CBS reference data, significant deviations from the 4B CCSD(T)/CBS reference data are associated with the bare revPBE-D3 functional.

On the other hand, Fig. 3 shows that noticeable differences are found in the (2B+3B)-XC RDFs calculated from analogous PIMD simulations carried out in the NPT ensemble (Fig. 3d). While the MB-pol RDFs remain in quantitative agreement with the experimental data, all four (2B+3B)-XC RDFs show significant deviations, with both (2B+3B)-revPBE-D3 and (2B+3B)-revPBE0-D3 predicting a slightly overstructured liquid and both (2B+3B)-B97M-rV and (2B+3B)-*ω*B97M-V predicting a slightly understructured liquid. These differences are directly related to the ability of the four XC functionals to correctly reproduce 2B and 3B energies (Fig. 1), and suggest that the four XC functionals effectively provide a phase diagram of water that is displaced in pressure and temperature compared to experiment, which is in line with previous studies indicating that popular XC functionals tend to predict a freezing point for water that is significantly larger than 273 K.^55^,^56^

Fig. 3b and e demonstrate that, when the original MB-pol 2B term is replaced by the corresponding term derived from 2B data calculated with the different XC functionals, with all other many-body terms of eqn (1) being represented as in MB-pol, the agreement between experimental and simulated RDFs deteriorates for all (2B)-XC PEFs, with the exception of (2B)-*ω*B97M-V. Specifically, both (2B)-revPBE-D3 and (2B)-revPBE0-D3 predict an unstructured liquid characterized by the collapse of the second solvation shell. While (2B)-B97M-rV still predicts a reasonable structure of liquid water when the PIMD simulations are carried out in the NVT ensemble, the agreement with the experimental RDF worsens in the NPT ensemble, with the simulated RDF barely showing a second solvation shell. Nearly quantitative agreement with the experimental RDF is instead predicted by PIMD simulations with the (2B)-*ω*B97M-V in both NVT and NPT ensembles. The poor performance by both (2B)-revPBE-D3 and (2B)-revPBE0-D3 can be traced back to the relatively large RMSDs associated with 2B energies calculated with these two XC functionals (Fig. 1). On the other hand, the different performance by (2B)-B97M-rV and (2B)-*ω*B97M-V PEFs suggests that an RMSD lower than ∼0.25 kcal mol^–1^ relative to the 2B CCSD(T)/CBS reference data adopted in the development of MB-pol and used in the correlation plots of Fig. 1 is required for a correct representation of 2B effects in liquid water.

When only the original MB-pol 3B term is replaced by the corresponding term derived from 3B data calculated with the different XC functionals, keeping all other many-body terms of eqn (1) as in MB-pol, Fig. 3c and f show that none of the (3B)-XC PEFs are able to correctly reproduce the experimental RDFs. In particular, (3B)-revPBE-D3, (3B)-revPBE0-D3 and, to a lesser extent, (3B)-B97M-rV predict an overstructured (*i.e.*, low density) liquid, a feature that is even more emphasized by PIMD simulations in the NPT ensemble. On the other hand, (3B)-*ω*B97M-V predicts an understructured (*i.e.*, high density) liquid. Combining the results obtained with the (2B)-XC and (3B)-XC PEFs, it is becomes evident that both (2B+3B)-revPBE-D3 and (2B+3B)-revPBE0-D3 achieve apparent agreement with the experimental RDFs through fortuitous error compensation in their representations of 2B and 3B interactions. Although similar conclusions can be drawn from the results obtained with the B97M-rV functional, it should be noted that the errors associated with the B97M-rV representations of 2B and 3B energies are significantly smaller than those associated with revPBE-D3 and revPBE0-D3, indicating that B97M-rV overall provides a more realistic description of liquid water. The case of *ω*B97M-V is completely different since this XC functional effectively provides CCSD(T)-level accuracy at the 2B level but is affected by nonnegligible errors in the representation of 3B energies. Thus the lack of error compensation in the representation of 2B and 3B interactions is the origin of the deviations between the experimental and (2B+3B)-*ω*B97M-V RDFs shown in Fig. 3a and d.

**Fig. 4:**
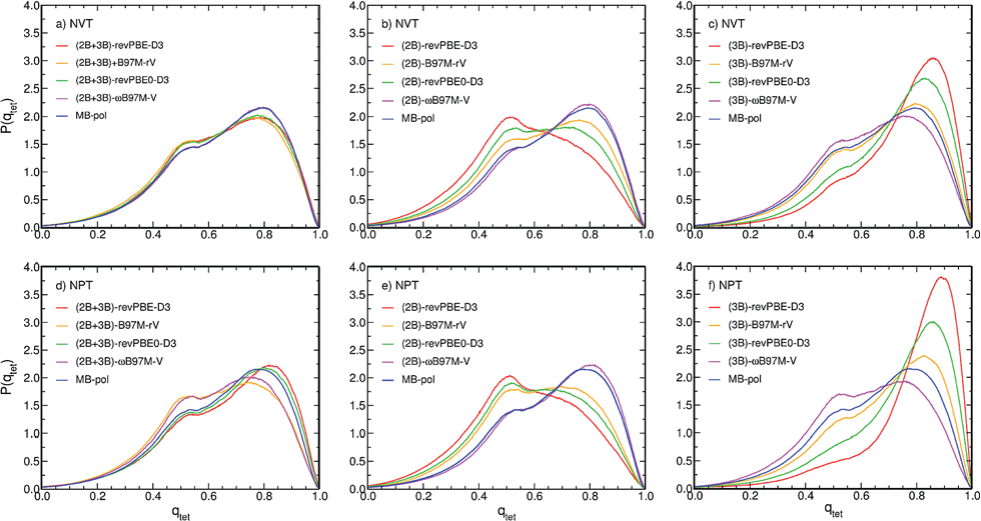
Comparison between normalized probability distributions of the tetrahedral order parameter, *P*(*q*_tet_), calculated from PIMD simulations carried out at 298.15 K in the NVT (panels a–c) and NPT (panels d–f) ensembles with the (2B+3B)-XC PEFs (panels a and d), (2B)-XC PEFs (panels b and e), and (3B)-XC PEFs (panels c and f) with XC = revPBE-D3 (red), B97M-rV (yellow), revPBE0-D3 (green), and *ω*B97M-V (magenta). Also shown in each panel are the corresponding distributions calculated from PIMD simulations with MB-pol.

The differences in the RDFs directly translate into differences in the local structure of liquid water as defined by the distribution of the tetrahedral order parameter, *P*(*q*_tet_), calculated from PIMD simulations with the different MB-XC PEFs, which represents an effective metric to determine the local structure of liquid water. Fig. 4a shows that all (2B+3B)-XC PEFs provide similar *P*(*q*_tet_) distributions as MB-pol when the PIMD simulations are carried out at the experimental density in the NVT ensemble. In contrast, the different RDFs obtained from PIMD simulations in the NPT ensemble lead to noticeably different *P*(*q*_tet_) distributions, with both (2B+3B)-B97M-rV and (2B+3B)-*ω*B97M-V providing a less tetrahedral liquid as indicated by the decrease of the main peak at *q*_tet_ = 0.8 and the increase of the peak at *q*_tet_ = 0.5. Instead both (2B+3B)-revPBE-D3 and (2B+3B)-revPBE0-D3 models predict a slightly more tetrahedral liquid, with the main peak shifted to higher q_tet_ values (Fig. 4d), as suggested by their overstructured RDFs.

Larger variations are found in the *P*(*q*_tet_) distributions calculated from PIMD simulations with the (2B)-XC and (3B)-XC PEFs in both NVT and NPT ensembles. Importantly, (2B)-*ω*B97M-V effectively provides the same *P*(*q*_tet_) distribution as MB-pol while (3B)-*ω*B97M-V predicts a slightly less tetrahedral liquid, which is a direct consequence of the different ability of *ω*B97M-V to reproduce 2B and 3B energies, as shown in Fig. 1. Directly following the trends observed in the corresponding RDFs, all other (2B)-XC and (3B)-XC PEFs respectively predict significantly less and more tetrahedral liquids. As already inferred from the analysis of Fig. 1 and 3, the differences in the local hydration structure predicted by the (2B)-XC and (3B)-XC PEFs are largely washed out when the simulations are carried out with the (2B+3B)-XC PEFs due to revPBE-D3, revPBE0-D3 and, to a lesser extent, B97M-rV benefiting from fortuitous error compensation in their representations of 2B and 3B energies.

## 4 Conclusions

Our study demonstrates that: (1) the local hydration structure of liquid water is primarily determined by the delicate interplay between 2B and 3B energies, which is further modulated by higher order (*i.e.*, larger than 3B) interactions; (2) a correct representation of 2B and 3B energies requires sub-chemical accuracy, which can be achieved by state-of-the-art many-body PEFs, such as MB-pol, rigorously derived from the MBE of eqn (1) where the individual terms are obtained at the CCSD(T)/CBS level of theory but is currently lacking in existing XC functionals that are commonly used in computer simulations of liquid water; (3) most of the XC functionals benefit from fortuitous error compensation in the representation of 2B and 3B energies, which may lead to apparent agreement with experiment for spatially uniform systems (*e.g.*, bulk water) but will likely result in inaccurate descriptions of non-uniform systems (*e.g.*, aqueous interfaces and electrolyte solutions) where error compensation may not be complete. While these results suggest that many-body PEFs, such as MB-pol, hold great promise for shedding light on the molecular origin of the anomalous behavior of liquid water as a function of temperature and pressure, they also warn that a definitive explanation of this behavior might require a level of accuracy in the representation of the underlying molecular interactions which may be out of reach for even the most sophisticated electronic structure methods currently available.

## Conflicts of interest

There are no conflicts to declare.

## Supplementary Material

Supplementary information
