# Supplementary material for: Low-order many-body interactions determine the local structure of liquid water
Source: Chem Sci. 2019 Jul 26;10(35):8211–8. doi: 10.1039/c9sc03291f (PMC6927411; doi:10.1039/c9sc03291f)
Supplement: Supplementary file 1 [file SC-010-C9SC03291F-s001.pdf]

Electronic Supplementary Material (ESI) for Chemical Science.
This journal is © The Royal Society of Chemistry 2019

Electronic Supplementary Information

Low-Order Many-Body Interactions Determine the Local Structure of Liquid Water

Marc Riera,[†] Eleftherios Lambros,[†] Thuong T. Nguyen,[†] Andreas W. Götz,[‡] and Francesco Paesani[*,†,¶,‡]

†*Department of Chemistry and Biochemistry, University of California, San Diego La Jolla, California 92093, United States*

‡*San Diego Supercomputer Center, University of California, San Diego La Jolla, California 92093, United States*

¶*Materials Science and Engineering, University of California, San Diego La Jolla, California 92093, United States*

E-mail: fpaesani@ucsd.edu

Contents

1 Many-body expansion of the total energy **S3**

2 MB-pol **S4**

3 Assessment of many-body interactions in water **S5**

4 Water simulations: O-H and H-H radial distribution functions **S12**

5 Water simulations: MB-XC PEFs versus bare XC functionals **S13**

References **S14**

1 Many-body expansion of the total energy

As mentioned in the main text, total energy (E_N) of a system containing N water molecules can be rigorously defined through the associated many-body expansion (MBE),[1]

$$\begin{aligned} E_N(1,\ldots,N) =& \sum_{i=1}^{N} E^{1B}(i) + \sum_{i<j}^{N} E^{2B}(i,j) \\ &+ \sum_{i<j<k}^{N} E^{3B}(i,j,k) + \ldots + E^{NB}(1,\ldots,N) \end{aligned} \tag{1}$$

where $E^{1B}(i)$ corresponds to the one-body (1B) energy required to deform the ith water molecule from its equilibrium geometry, and $E^{nB}(i,j,\ldots,n)$ are the n-body (nB) energies defined recursively as

$$\begin{aligned} E^{nB}(1,\ldots,n) =& E_n(1,\ldots,n) - \sum_{i} E^{1B}(i) - \sum_{i<j} E^{2B}(i,j) - \ldots \\ & \ldots - \sum_{i<j<\ldots<n-1} E^{(n-1)B}(i,j,\ldots,(n-1)) \end{aligned} \tag{2}$$

It has been shown that Eq. 1 converges rapidly for water, with the 2B and 3B terms contributing, on average, ∼80% and 15–20%, respectively, and all remaining higher-body terms contributing ∼1%.[1–3] Exploiting the rapid convergence of the MBE for water, SAMBA can be used to provide highly accurate interaction energies for water clusters through the application of progressively lower-level electronic structure methods to represent subsequently higher-body terms of the MBE.[4]

Tremendous progress has been made in the past years in devising mathematical functions that can accurately represent multidimensional potential energy surfaces with a large number of degrees of freedom.[5–7] This has led to the development of several many-body (MB) potential energy functions (PEFs), including CC-pol,[8–10] WHBB,[11–13] HBB2-pol[14,15] and MB-pol[16–18]). The interested reader is referred to Ref. 19 for a recent review of MB PEFs. Among existing many-body PEFs, it has been shown that MB-pol provides an accurate rep-

resentation of the molecular properties of water from the gas to the condensed phase,[20,21] correctly predicting the vibration-rotation tunneling spectrum of the dimer,[16] the relative stability and vibrational spectra of small water clusters,[22,23] the structural, thermodynamic, and dynamical properties[18] as well as the IR and Raman spectra of liquid water,[24–27] the vibrational sum-frequency generation (vSFG) spectrum of the air/water interface,[28,29] and the energetics[30] and vibrational spectra[31,32] of different ice phases.

2 MB-pol

MB-pol was rigorously derived from Eq. 1 and includes explicit 1B, 2B, and 3B terms, with all higher-order contributions being accounted for in a mean field sense through a classical representation of N-body induction.[33] Each MB-pol water molecule is represented by six sites, with three sites corresponding to the oxygen and two hydrogen atoms, two fictitious sites located symmetrically along the directions of the oxygen lone-pairs, and an additional M-site located along the HOH bisector (see Ref. 33 for details). The MB-pol 1B term corresponds to the monomer PEF developed by Partridge and Schwenke[34] which is also associated with a nonlinear dipole moment surface represented by geometry dependent point charges on the two hydrogen atoms (positive charges) and M-site (balancing negative charge). The molecular polarizability tensor of an isolated water molecule is represented by isotropic atomic polarizabilities located on the oxygen and hydrogen atoms. The MB-pol 2B term is represented through a combination of permanent and induced electrostatics, and dispersion energy, which are supplemented with a multidimensional term correcting for deficiencies associated with a purely classical representation of intermolecular interactions at short range (e.g., charge transfer, charge penetration and Pauli repulsion).[35] Both 2B permanent electrostatics and induction are represented by a modified Thole-type expression derived from the TTM4-F model,[36] while the dispersion energy is expressed through a rigorous fit to the asymptotic *ab initio* reference energy as originally introduced by the CC-pol

PEF.[8] Similarly, MB-pol represents 3B interactions by combining a classical 3B induction term with a multidimensional short-range function. The short-range 2B and 3B multidimensional functions are expressed by 4th-degree permutationally invariant polynomials (PIPs)[5] in variables that are functions of the distances between all six molecular sites. The coefficients of both 2B and 3B PIPs were optimized to reproduce 2B and 3B energies calculated at the CCSD(T)/CBS level of theory for approximately 42,508 and 12,347 dimers and trimers, respectively (see Refs. 33 and 17 for specific details).

3 Assessment of many-body interactions in water

The low-lying isomers of $(H_2O)_n$ clusters, with $n = 2 - 6$, considered in the analysis of many-body interactions are shown in Fig. S1. Cluster geometries for $n = 2 - 3$ where taken from Ref. 21, those for $n = 4 - 5$ were taken from Ref. 37, and those for $n = 6$ were taken from Ref. 38.

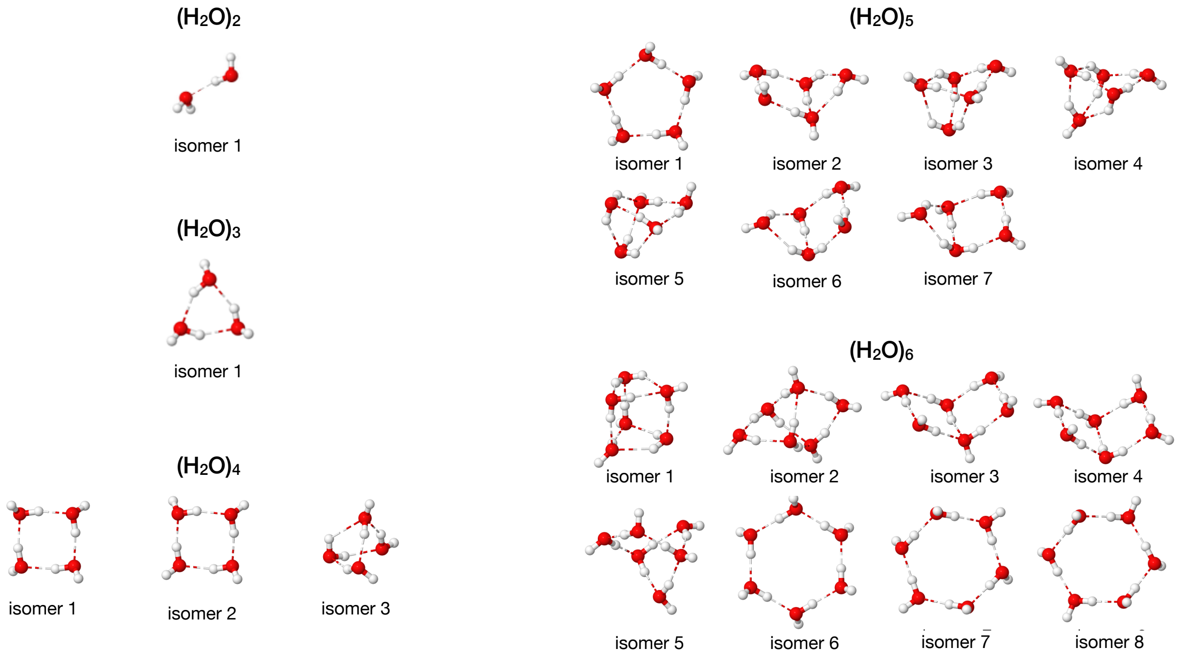

Figure S1: Geometries of the low-lying isomers of $(H_2O)_n$ clusters, with $n = 2 - 6$, used in the analysis of interaction and many-body energies in Section 5.

To assess the accuracy of revPBE-D3, B97M-rV, revPBE0-D3, ωB97M-V and MB-pol in representing many-body interactions in water, Figs. S2−S6 show both interaction energies (left panels) and many-body energies (right panels) calculated for the low-lying isomers of the water clusters shown in Fig. S1 using all four XC functionals and MB-pol compared with the corresponding values calculated, within SAMBA, at the CCSD(T)/CBS level of theory. This analysis shows that both revPBE-D3 and revPBE0-D3 exhibit relatively large deviations from the CCSD(T)/CBS reference values at the 2B levels, while nonnegligible differences are still present at both 3B and 4B levels. Although these deviations are significantly reduced in calculations with B97M-rV and ωB97M-V, they are still nonnegligible at the 2B and 4B levels for B97M-rV and at the 3B level for ωB97M-V. Noting that "chemical accuracy" is often defined as a deviation smaller than 1.0 kcal/mol relative to the CCSD(T)/CBS value, MB-pol achieves sub-chemical accuracy for both interaction energies and individual many-body terms for all clusters, from the dimer to the hexamer, providing in all cases the closest agreement with the CCSD(T)/CBS reference data.

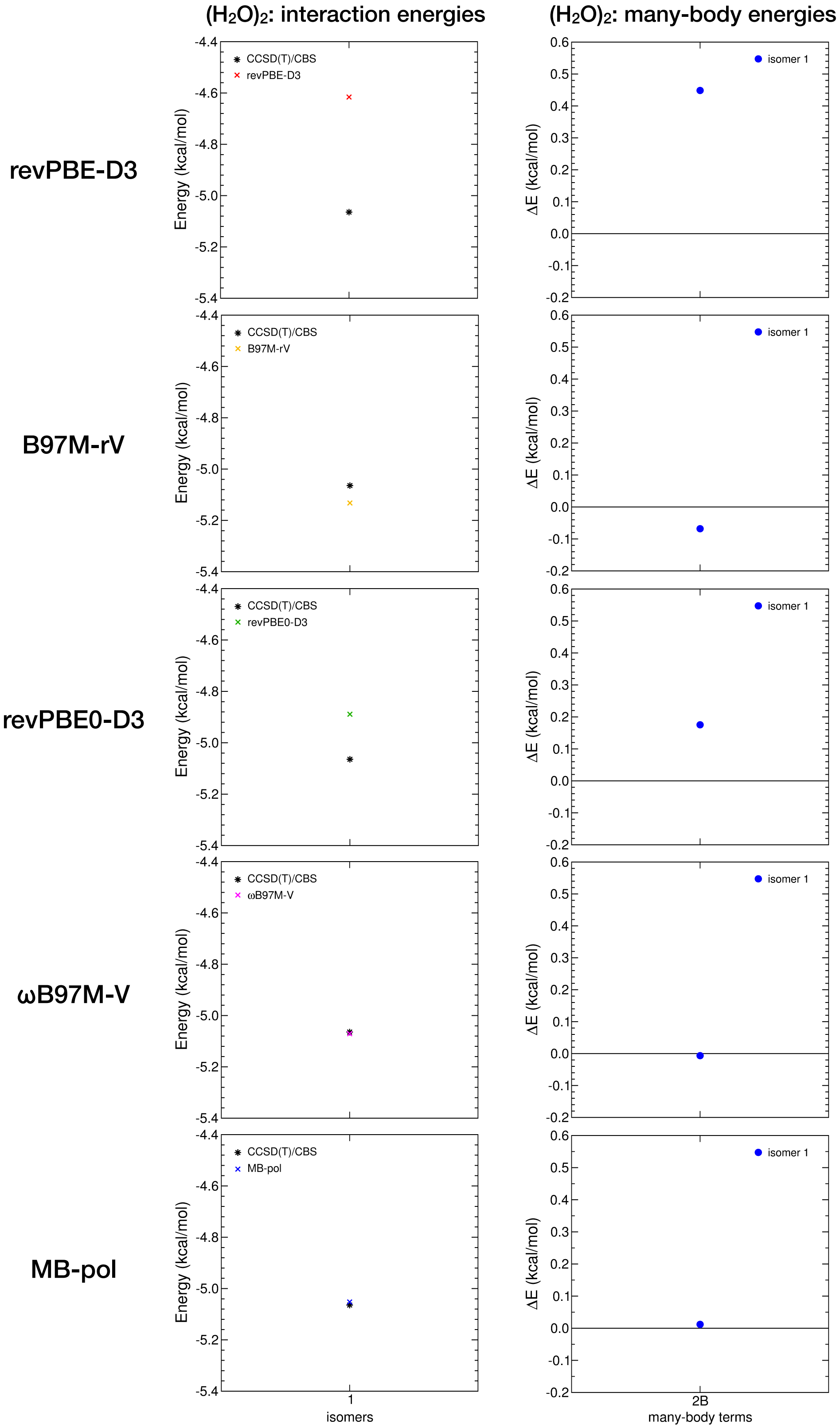

Figure S2: Interaction energies (left) and deviations relative to CCSD(T)/CBS reference data, $\Delta E = E_{model} - E_{CCSD(T)}$ (right), for individual many-body energies, E^{nB} in Eq. 2, calculated with revPBE-D3, B97M-rV, revPBE0-D3, ωB97M-V and MB-pol for the low-lying isomer of the $(H_2O)_2$ cluster shown in Fig. S1.

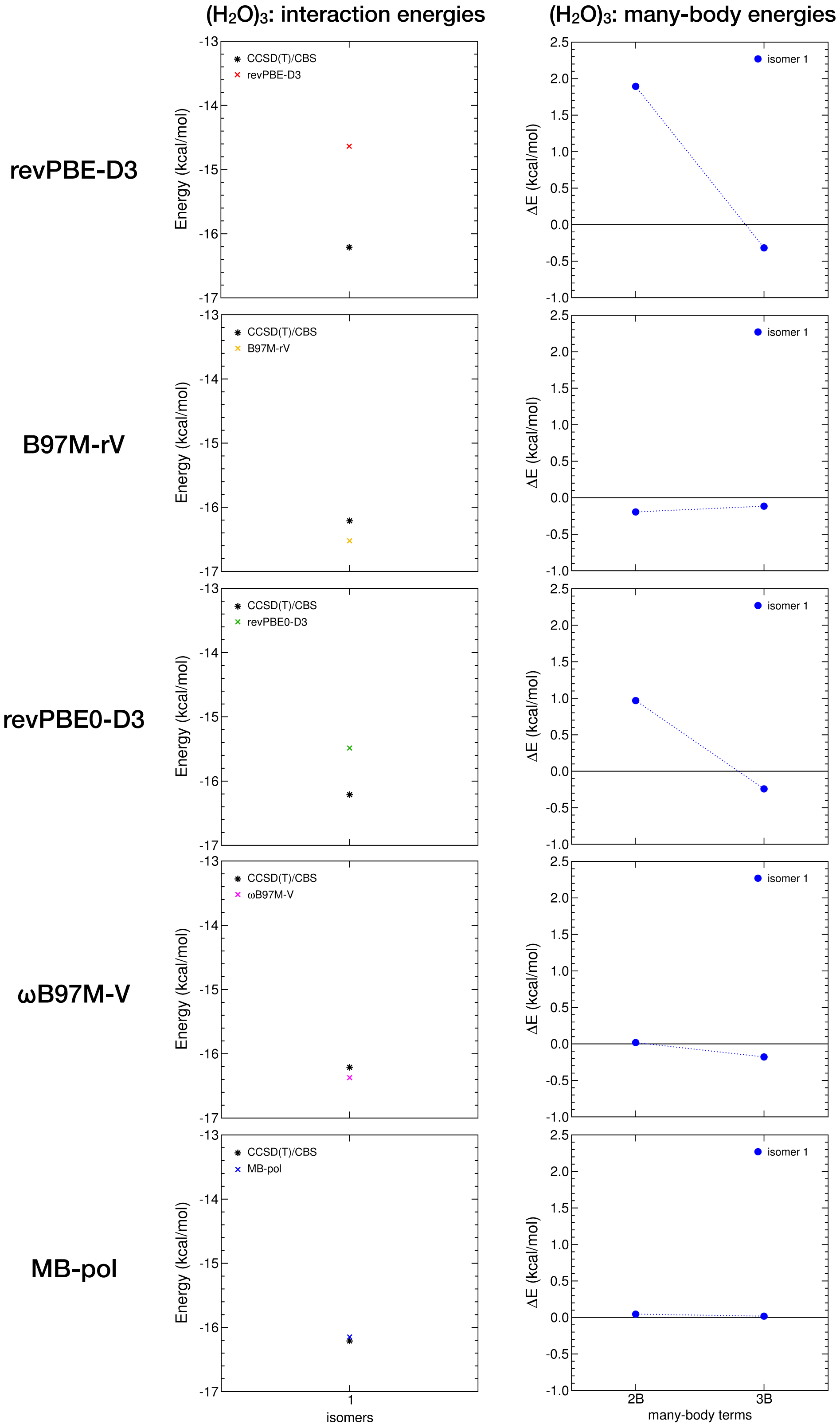

Figure S3: Interaction energies (left) and deviations relative to CCSD(T)/CBS reference data, $\Delta E = E_{model} - E_{CCSD(T)}$ (right), for individual many-body energies, E^{nB} in Eq. 2, calculated with revPBE-D3, B97M-rV, revPBE0-D3, ωB97M-V and MB-pol for the low-lying isomer of the $(H_2O)_3$ cluster shown in Fig. S1.

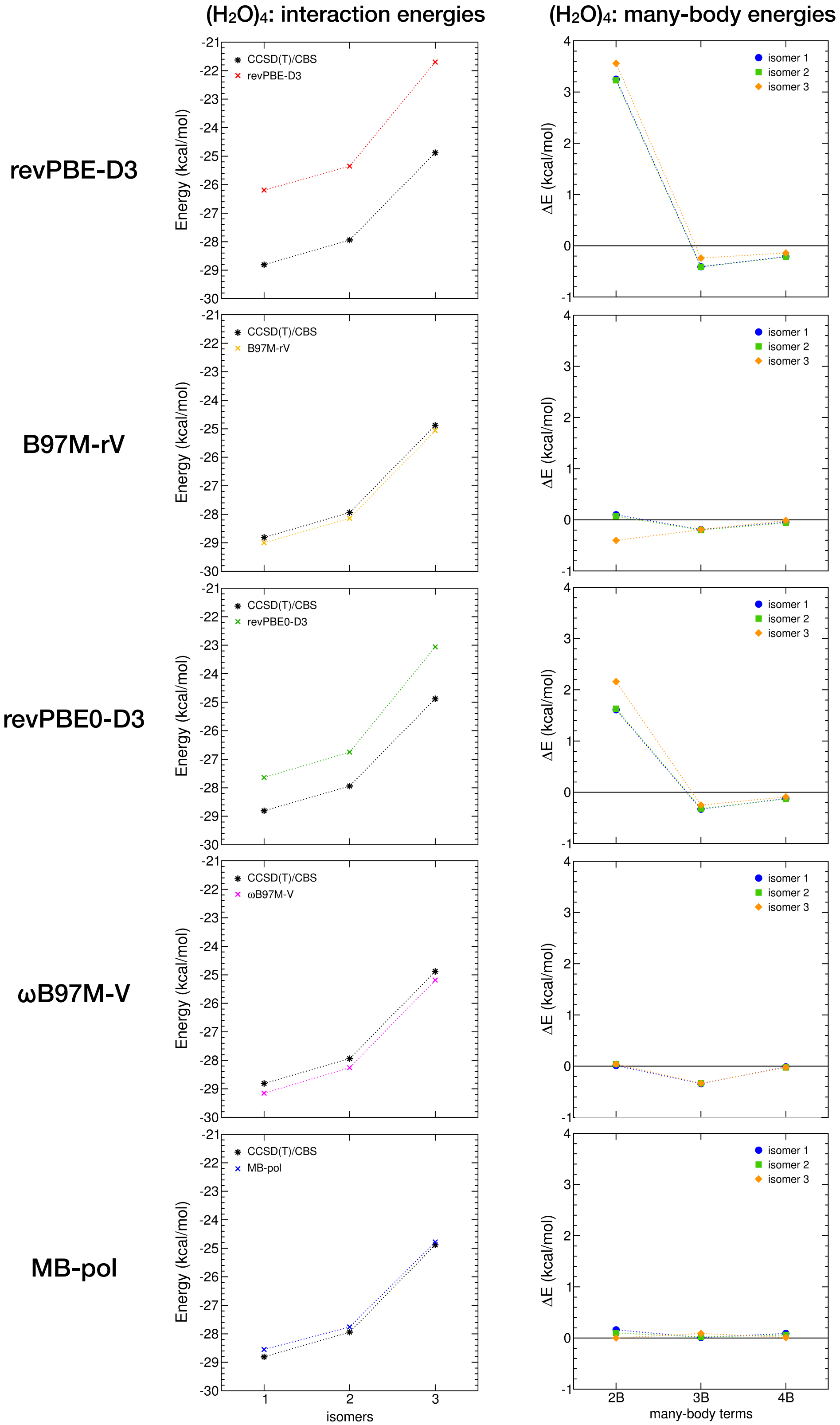

Figure S4: Interaction energies (left) and deviations relative to CCSD(T)/CBS reference data, $\Delta E = E_{model} - E_{CCSD(T)}$ (right), for individual many-body energies, E^{nB} in Eq. 2, calculated with revPBE-D3, B97M-rV, revPBE0-D3, ωB97M-V and MB-pol for the low-lying isomers of the $(H_2O)_4$ cluster shown in Fig. S1.

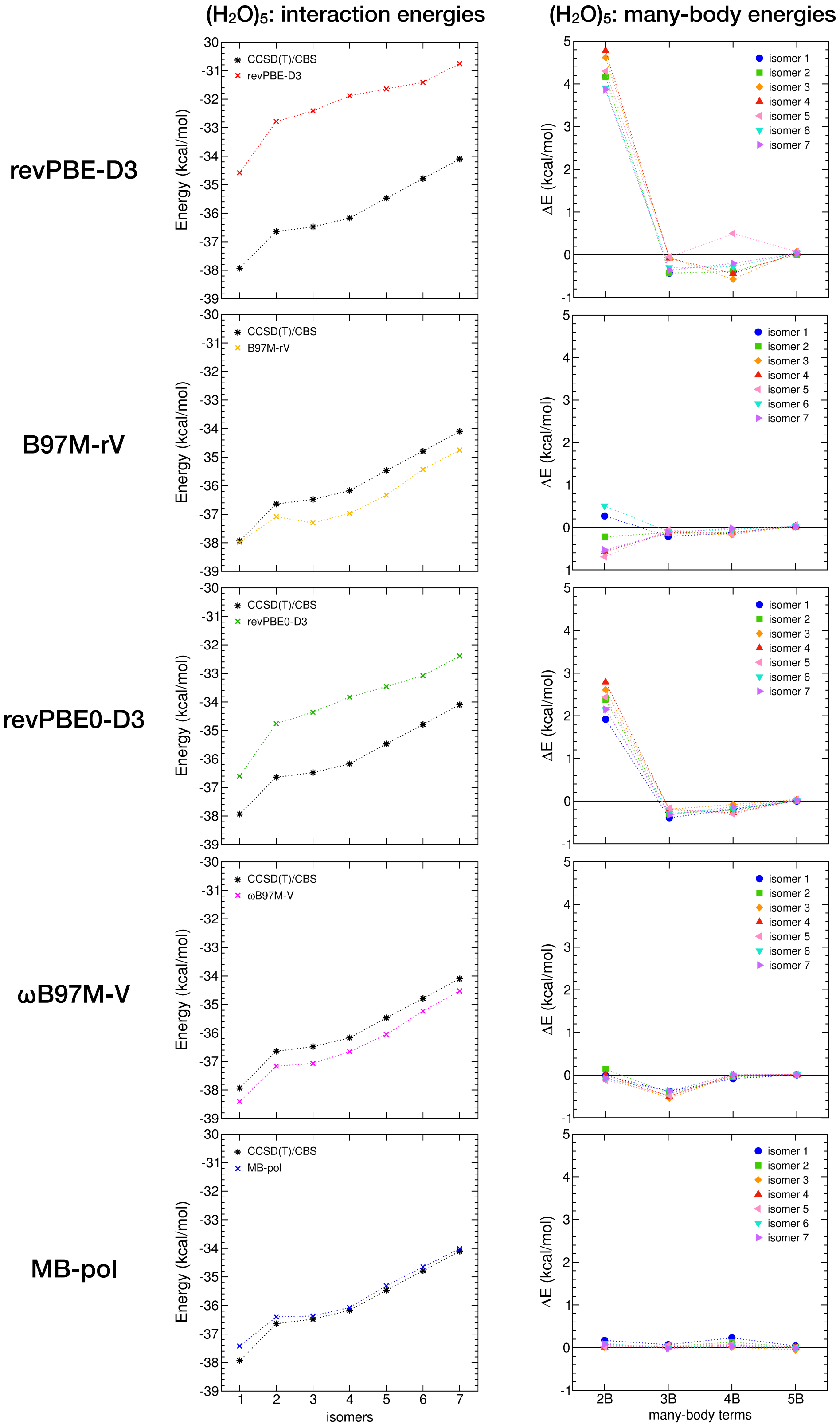

Figure S5: Interaction energies (left) and deviations relative to CCSD(T)/CBS reference data, $\Delta E = E_{model} - E_{CCSD(T)}$ (right), for individual many-body energies, E^{nB} in Eq. 2, calculated with revPBE-D3, B97M-rV, revPBE0-D3, ωB97M-V and MB-pol for the low-lying isomers of the $(H_2O)_5$ cluster shown in Fig. S1.

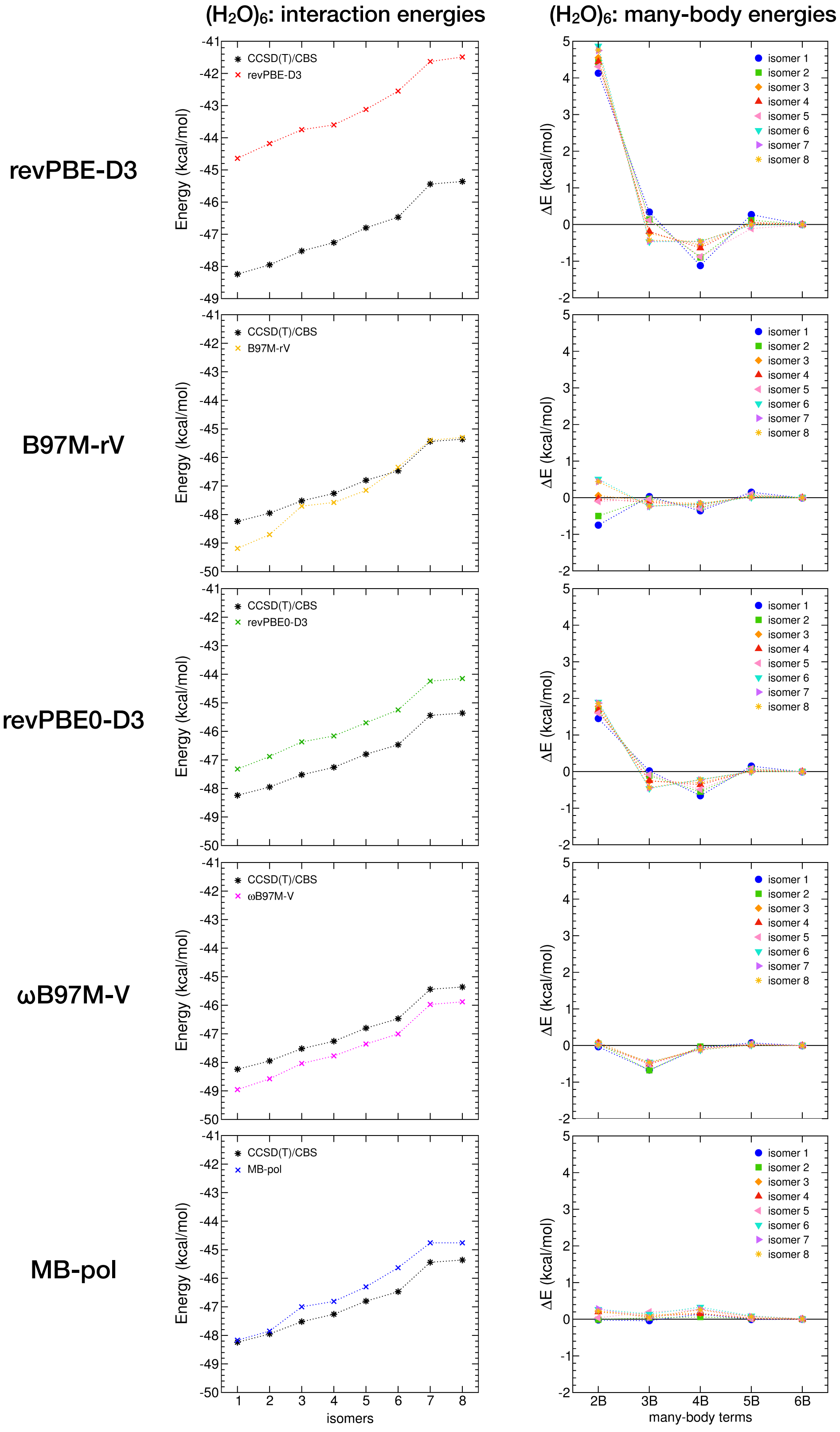

Figure S6: Interaction energies (left) and deviations relative to CCSD(T)/CBS reference data, $\Delta E = E_{model} - E_{CCSD(T)}$ (right), for individual many-body energies, E^{nB} in Eq. 2, calculated with revPBE-D3, B97M-rV, revPBE0-D3, ωB97M-V and MB-pol for the low-lying isomers of the $(H_2O)_6$ cluster shown in Fig. S1.

4 Water simulations: O-H and H-H radial distribution functions

To complete the analysis presented in the main text, Figs. S7 and S8 show comparisons between the oxygen–hydrogen (O-H) and hydrogen–hydrogen (H-H) radial distribution functions (RDFs) calculated from PIMD simulations carried out in both NVT and NPT ensembles using each of the (MB)-XC PEFs introduced in this study. Also shown are the corresponding experimental[39,40] and MB-pol[18,21,26] RDFs. Similar conclusions to those discussed in the main text can also be drawn from the analysis of the O-H and H-H RDFs, with all (MB)-XC PEFs benefiting from error compensation in their representation of 2B and 3B interactions and providing relatively better agreement with the experimental data when the PIMD simulations are carried out in the NVT ensemble.

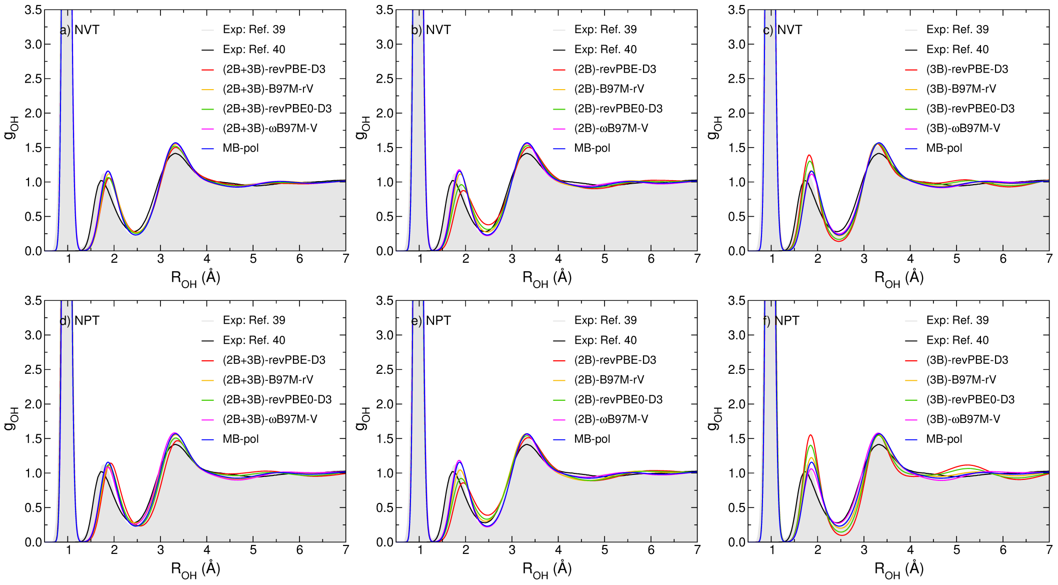

Figure S7: Comparison between experimental and theoretical oxygen–hydrogen (O-H) radial distribution functions (RDFs) of liquid water at ambient conditions. The experimental data are taken from Refs. 39 (gray area) and 40 (black line). The theoretical RDFs were calculated from PIMD simulations carried out at 298.15 K in the NVT (top panels) and NPT (bottom panels) ensembles with the (2B+3B)-XC PEFs (left panels), (2B)-XC PEFs (middle panels), and (3B)-XC PEFs (right panels) with XC = revPBE-D3 (red), B97M-rV (yellow), revPBE0-D3 (green), and ωB97M-V (magenta). Also shown in each panel are the corresponding RDFs calculated from PIMD simulations with MB-pol.[18,21,26]

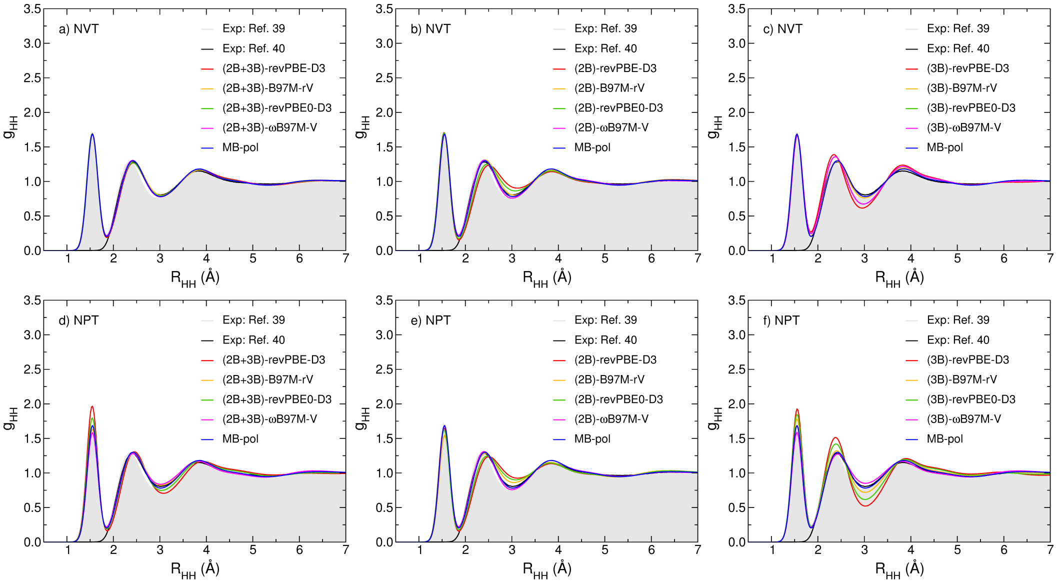

Figure S8: Comparison between experimental and theoretical hydrogen–hydrogen (H-H) radial distribution functions (RDFs) of liquid water at ambient conditions. The experimental data are taken from Refs. 39 (gray area) and 40 (black line). The theoretical RDFs were calculated from PIMD simulations carried out at 298.15 K in the NVT (top panels) and NPT (bottom panels) ensembles with the (2B+3B)-XC PEFs (left panels), (2B)-XC PEFs (middle panels), and (3B)-XC PEFs (right panels) with XC = revPBE-D3 (red), B97M-rV (yellow), revPBE0-D3 (green), and ωB97M-V (magenta). Also shown in each panel are the corresponding RDFs calculated from PIMD simulations with MB-pol.[18,21,26]

5 Water simulations: MB-XC PEFs versus bare XC functionals

To assess the ability of the MB-XC PEFs introduced in this study to reproduce results obtained fully *ab initio* using the corresponding XC functionals, the oxygen-oxygen (O-O) radial distribution functions (RDFs) obtained from PIMD simulations carried out with the (2B+3B)-revPBE-D3, (2B+3B)-revPBE0-D3, and (2B+3B)-B97M-rV PEFs at 298.15 K in the NVT ensemble are compared in Fig. S9 with the corresponding RDFs obtained in Refs. 41 and 42 from *ab initio* PIMD simulations with the bare revPBE-D3, revPBE0-D3, and B97M-rV functionals. In all cases, good agreement is found between the two sets of RDFs, which is effectively quantitative in the case of revPBE0-D3 and B97M-rV. This comparison demonstrates that the (2B+3B)-XC PEFs faithfully mimic the corresponding XC

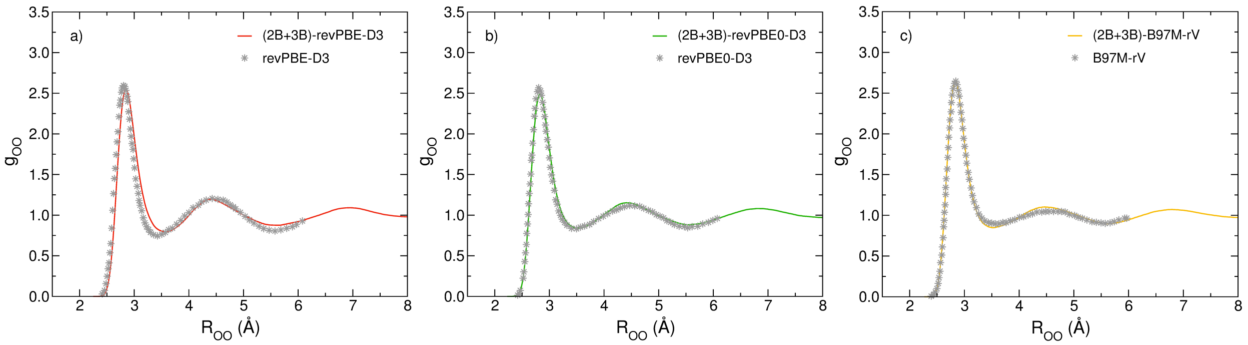

Figure S9: Comparison between oxygen-oxygen radial distribution functions (RDFs), g_{OO}, of liquid water at 298.15 K calculated from PIMD simulations carried out with the (2B+3B)-revPBE-D3 (panel a), (2B+3B)-revPBE0-D3 (panel b), and (2B+3B)-B97M-rV (panel c) PEFs and the corresponding RDFs calculated from PIMD simulations with the bare revPBE-D3, revPBE0-D3, and B97M-rV functionals in Refs. 41 and 42. Both sets of PIMD simulations were carried out in the NVT ensemble at the experimental water density, although 256 molecules were used in simulations with (2B+3B)-revPBE-D3, (2B+3B)-revPBE0-D3, and (2B+3B)-B97M-rV, while 64 molecules were used in simulations with the bare XC functionals.

functionals, indicating that the MB-pol functional form provides an efficient, yet accurate, computational route for developing analytical PEFs that allow for reproducing results of fully *ab initio* simulations, at a fraction of the cost associated with the latter. It should also be noted that the small differences seen in the comparison between the O-O RDF calculated with the (2B+3B)-revPBE-D3 PEF and the corresponding results from *ab initio* simulations with the bare revPBE-D3 functional are likely due to differences in how (2B+3B)-revPBE and revPBE-D3 represent 4B energies. While in (2B+3B)-revPBE the 4B energies are described as in MB-pol which, as shown in Figs. S4-S6, provides excellent agreement with the CCSD(T)/CBS reference data, significant deviations from the 4B CCSD(T)/CBS reference data are associated with the bare revPBE-D3 functional.

References

(1) Hankins, D.; Moskowitz, J.; Stillinger, F. Water Molecule Interactions. *J. Chem. Phys.* **1970**, *53*, 4544–4554.

(2) Del Bene, J.; Pople, J. Intermolecular Energies of Small Water Polymers. *Chem. Phys. Lett.* **1969**, *4*, 426–428.

(3) Kim, K.; Dupuis, M.; Lie, G.; Clementi, E. Revisiting Small Clusters of Water Molecules. *Chem. Phys. Lett.* **1986**, *131*, 451–456.

(4) Góra, U.; Podeszwa, R.; Cencek, W.; Szalewicz, K. Interaction Energies of Large Clusters from Many-Body Expansion. *J. Chem. Phys.* **2011**, *135*, 224102.

(5) Braams, B. J.; Bowman, J. M. Permutationally Invariant Potential Energy Surfaces in High Dimensionality. *Int. Rev. Phys. Chem.* **2009**, *28*, 577–606.

(6) Bartók, A. P.; Payne, M. C.; Kondor, R.; Csányi, G. Gaussian Approximation Potentials: The accuracy of Quantum Mechanics, Without the Electrons. *Phys. Rev. Lett.* **2010**, *104*, 136403.

(7) Behler, J. Perspective: Machine Learning Potentials for Atomistic Simulations. *J. Chem. Phys.* **2016**, *145*, 170901.

(8) Bukowski, R.; Szalewicz, K.; Groenenboom, G. C.; van der Avoird, A. Predictions of the Properties of Water from First Principles. *Science* **2007**, *315*, 1249–1252.

(9) Bukowski, R.; Szalewicz, K.; Groenenboom, G. C.; van der Avoird, A. Polarizable Interaction Potential for Water from Coupled Cluster Calculations. I. Analysis of Dimer Potential Energy Surface. *J. Chem. Phys.* **2008**, *128*, 094313.

(10) Bukowski, R.; Szalewicz, K.; Groenenboom, G. C.; van der Avoird, A. Polarizable Interaction Potential for Water from Coupled Cluster Calculations. II. Applications to Dimer Spectra, Virial Coefficients, and Simulations of Liquid Water. *J. Chem. Phys.* **2008**, *128*, 094314.

(11) Wang, Y.; Shepler, B. C.; Braams, B. J.; Bowman, J. M. Full-Dimensional, *Ab Initio*

Potential Energy and Dipole Moment Surfaces for Water. *J. Chem. Phys.* **2009**, *131*, 054511.

(12) Wang, Y.; Huang, X.; Shepler, B. C.; Braams, B. J.; Bowman, J. M. Flexible, *Ab Initio* Potential, and Dipole Moment Surfaces for Water. I. Tests and Applications for Clusters up to the 22-mer. *J. Chem. Phys.* **2011**, *134*, 094509.

(13) Wang, Y.; Bowman, J. M. *Ab Initio* Potential and Dipole Moment Surfaces for Water. II. Local-Monomer Calculations of the Infrared Spectra of Water Clusters. *J. Chem. Phys.* **2011**, *134*, 154510.

(14) Babin, V.; Medders, G. R.; Paesani, F. Toward a Universal Water Model: First Principles Simulations from the Dimer to the Liquid Phase. *J. Phys. Chem. Lett.* **2012**, *3*, 3765–3769.

(15) Medders, G. R.; Babin, V.; Paesani, F. A Critical Assessment of Two-Body and Three-Body Interactions in Water. *J. Chem. Theory Comput.* **2013**, *9*, 1103–1114.

(16) Babin, V.; Paesani, F. The Curious Case of the Water Hexamer: Cage vs. Prism. *Chem. Phys. Lett.* **2013**, *580*, 1 – 8.

(17) Babin, V.; Medders, G. R.; Paesani, F. Development of a "First Principles" Water Potential with Flexible Monomers. II: Trimer Potential Energy Surface, Third Virial Coefficient, and Small Clusters. *J. Chem. Theory Comput.* **2014**, *10*, 1599–1607.

(18) Medders, G. R.; Babin, V.; Paesani, F. Development of a "First-Principles" Water Potential with Flexible Monomers. III. Liquid Phase Properties. *J. Chem. Theory Comput.* **2014**, *10*, 2906–2910.

(19) Cisneros, G. A.; Wikfeldt, K. T.; Ojamäe, L.; Lu, J.; Xu, Y.; Torabifard, H.; Bartók, A. P.; Csányi, G.; Molinero, V.; Paesani, F. Modeling Molecular Interactions in

Water: From Pairwise to Many-Body Potential Energy Functions. *Chem. Rev.* **2016**, *116*, 7501–7528.

(20) Paesani, F. Getting the Right Answers for the Right Reasons: Toward Predictive Molecular Simulations of Water with Many-Body Potential Energy Functions. *Acc. Chem. Res.* **2016**, *49*, 1844–1851.

(21) Reddy, S. K.; Straight, S. C.; Bajaj, P.; Huy Pham, C.; Riera, M.; Moberg, D. R.; Morales, M. A.; Knight, C.; Götz, A. W.; Paesani, F. On the Accuracy of the Mb-Pol Many-Body Potential for Water: Interaction Energies, Vibrational Frequencies, and Classical Thermodynamic and Dynamical Properties from Clusters to Liquid Water and Ice. *J. Chem. Phys.* **2016**, *145*, 194504.

(22) Richardson, J. O.; Pérez, C.; Lobsiger, S.; Reid, A. A.; Temelso, B.; Shields, G. C.; Kisiel, Z.; Wales, D. J.; Pate, B. H.; Althorpe, S. C. Concerted hydrogen-bond breaking by quantum tunneling in the water hexamer prism. *Science* **2016**, *351*, 1310–1313.

(23) Cole, W. T. S.; Farrell, J. D.; Wales, D. J.; Saykally, R. J. Structure and Torsional Dynamics of the Water Octamer from THz Laser Spectroscopy Near 215 μm. *Science* **2016**, *352*, 1194–1197.

(24) Medders, G. R.; Paesani, F. Infrared and Raman Spectroscopy of Liquid Water through "First-Principles" Many-Body Molecular Dynamics. *J. Chem. Theory Comput.* **2015**, *11*, 1145–1154.

(25) Straight, S. C.; Paesani, F. Exploring Electrostatic Effects on the Hydrogen Bond Network of Liquid Water through Many-Body Molecular Dynamics. *J. Phys. Chem. B* **2016**, *120*, 8539.

(26) Reddy, S. K.; Moberg, D. R.; Straight, S. C.; Paesani, F. Temperature-Dependent Vibrational Spectra and Structure of Liquid Water from Classical and Quantum Simulations with the MB-pol Potential Energy Function. *J. Chem. Phys.* **2017**, *147*, 244504.

(27) Hunter, K. M.; Shakib, F. A.; Paesani, F. Disentangling Coupling Effects in the Infrared Spectra of Liquid Water. *J. Phys. Chem. B* **2018**, *122*, 10754–10761.

(28) Medders, G. R.; Paesani, F. Dissecting the Molecular Structure of the Air/Water Interface from Quantum Simulations of the Sum- Frequency Generation Spectrum. *J. Am. Chem. Soc.* **2016**, *138*, 3912–3919.

(29) Moberg, D. R.; Straight, S. C.; Paesani, F. Temperature Dependence of the Air/Water Interface Revealed by Polarization Sensitive Sum-Frequency Generation Spectroscopy. *J. Phys. Chem. B* **2018**, *122*, 4356–4365.

(30) Pham, C. H.; Reddy, S. K.; Chen, K.; Knight, C.; Paesani, F. Many-Body Interactions in Ice. *J. Chem. Theory Comput.* **2017**, *13*, 1778–1784.

(31) Moberg, D. R.; Straight, S. C.; Knight, C.; Paesani, F. Molecular Origin of the Vibrational Structure of Ice Ih. *J. Phys. Chem. Lett.* **2017**, *8*, 2579–2583.

(32) Moberg, D. R.; Sharp, P. J.; Paesani, F. Molecular-Level Interpretation of Vibrational Spectra of Ordered Ice Phases. *J. Phys. Chem. B* **2018**, *122*, 10572–10581.

(33) Babin, V.; Leforestier, C.; Paesani, F. Development of a "First Principles" Water Potential with Flexible Monomers: Dimer Potential Energy Surface, VRT Spectrum, and Second Virial Coefficient. *J. Chem. Theory Comput.* **2013**, *9*, 5395–5403.

(34) Partridge, H.; Schwenke, D. W. The Determination of an Accurate Isotope Dependent Potential Energy Surface for Water from Extensive Ab Initio Calculations and Experimental Data. *J. Chem. Phys.* **1997**, *106*, 4618–4639.

(35) Paesani, F. Water: Many-Body Potential from First Principles (From the Gas to the Liquid Phase). *Handbook of Materials Modeling: Methods: Theory and Modeling* **2018**, 1–25.

(36) Burnham, C. J.; Anick, D. J.; Mankoo, P. K.; Reiter, G. F. The Vibrational Proton Potential in Bulk Liquid Water and Ice. *J. Chem. Phys.* **2008**, *128*, 154519.

(37) Temelso, B.; Archer, K. A.; Shields, G. C. Benchmark Structures and Binding Energies of Small Water Clusters with Anharmonicity Corrections. *J. Phys. Chem. A* **2011**, *115*, 12034–12046.

(38) Bates, D. M.; Tschumper, G. S. CCSD(T) Complete Basis Set Limit Relative Energies for Low-Lying Water Hexamer Structures. *J. Phys. Chem. A* **2009**, *113*, 3555–3559.

(39) Soper, A. The radial distribution functions of water and ice from 220 to 673 K and at pressures up to 400 MPa. *Chem. Phys.* **2000**, *258*, 121–137.

(40) Soper, A.; Benmore, C. Quantum differences between heavy and light water. *Phys. Rev. Lett.* **2008**, *101*, 065502.

(41) Marsalek, O.; Markland, T. E. Quantum Dynamics and Spectroscopy of Ab Initio Liquid Water: The Interplay of Nuclear and Electronic Quantum Effects. *J. Phys. Chem. Lett.* **2017**, *8*, 1545–1551.

(42) Ruiz Pestana, L.; Marsalek, O.; Markland, T. E.; Head-Gordon, T. The Quest for Accurate Liquid Water Properties from First Principles. *J. Phys. Chem. Lett.* **2018**, *9*, 5009–5016.
